# Early satisfactory results of percutaneous repair in neglected achilles tendon rupture

**DOI:** 10.1186/s12891-023-06561-0

**Published:** 2023-06-02

**Authors:** Mostafa Elsebai Hammad, Aly Maher Fayed, Mostafa Ahmed Ayoub, Ali Mahmoud Emran

**Affiliations:** Department of Orthopaedic surgery, Faculty of medicine, Tanta University, Hassan Radwan st. Tanta city, Gharbia governorate, Tanta city, Al-gharbia governorate, ZIP:31511 Egypt

**Keywords:** Achilles tendon, Minimally invasive, Neglected rupture, Percutaneous repair

## Abstract

**Purpose:**

This investigation aimed to study the outcome of percutaneous repair of Achilles tendon ruptures regarding patient-reported and objective outcomes.

**Methods:**

This is a retrospective review of a cohort of patients (n = 24) who underwent percutaneous repair of neglected Achilles rupture in the period between 2013 and 2019. Included patients were adults with closed injuries, presented 4–10 weeks after rupture, with intact deep sensation. All underwent clinical examination, X-rays to exclude bony injury and MRI for diagnosis confirmation. All underwent percutaneous repair by the same surgeon, using the same technique and rehabilitation protocol. The postoperative assessment was done subjectively using ATRS and AOFAS score and objectively using a percentage of heel rise comparison to the normal side and calf circumference difference.

**Results:**

The mean follow-up period was 14.85 months ± 3 months. Average AOFAS scores at 6,12 months were 91 and 96, respectively, showing statistically significant improvement from pre-op level (P < 0.001). Percentage of heel rise on the affected side and calf circumference showed statistically significant improvement over the 12 month follow up period (P < 0.001). Superficial infection was reported in two patients (8.3%), and two cases reported transient sural nerve neuritis.

**Conclusion:**

Percutaneous repair of neglected Achilles rupture using the index technique proved a satisfactory patient-reported and objective measurement at a one-year follow-up. With only minor transient complications.

## Introduction

The Achilles tendon is formed of a tendinous confluence of the soleus and the two heads of the gastrocnemius muscles, and attaches to the calcaneal tuberosity. It is the thickest tendon in the human body (5–7 mm) and can stretch up to 4% before being damaged. The posterior surace of the achilles is less vascularized than the anterior surface, and the vascular water-shed area ( 5 to 7 cm from the calcaneal insertion) is the most common site of tendon rupture [1, 2].

Rupture of the Achilles tendon is a common injury, with incidence ranging from 11 to 38 in 100,000 [3, 4]. It is the most common tendon to be injured in the lower limb, particularly during sports [5]. Although the diagnosis is relatively easy clinically, 10–25% of Tendoachilles injuries are missed [6].

For an injury to be considered neglected, four weeks delay in diagnosis is considered in most literature [7–9]. Management of such cases is still a matter of debate. However, surgery is considered in most instances to avoid the risks of prolonged immobilization, residual weakness, and re-rupture [5, 8, 10, 11].

Several surgical methods for chronic/neglected Achilles rupture have been proposed, such as conventional end-to-end repair using Bunnell or Kessler sutures. However, these methods can result in excess tension, which may compromise the repair. Using fascia or tendon grafts (from plantaris, peroneus brevis, flexor hallucis longus or free semitendinosus transfer) may reinforce the repair; the gap could be managed by gastrocnemius turn down flaps, V-Y sliding flap or wrapping a remaining defect with fascia lata graft [5, 8, 9, 11].

Furthermore, most conventional surgical techniques require long skin incisions prone to infection, necrosis of skin edges, and tendon adhesions [12]. Some authors have incorporated skin flap techniques to facilitate closure following reconstructive procedures for the Achilles tendon [11].

Generally, minimally invasive techniques have less operative time, are more cosmetically preferred, and allow other procedures in failed repair. Reports in literature regarding percutaneous repair in neglected rupture of the Achilles tendon are limited in number, however, the reported results were encouraging [9, 13, 14]. The objective of this study was to assess the subjective and objective outcomes of a modified percutaneous repair technique for neglected total Achilles tendon ruptures.

## Patients and methods

We retrospectively analyzed the pre-collected data of patients presenting between August 2013 – August 2019 with neglected rupture of Achilles tendon. Inclusion criteria were closed Achilles tendon rupture in adults presented four to ten weeks after injury with intact deep sensation. Patients with acute rupture (< 4 weeks), open injury, bony avulsions of the Achilles tendon, re-rupture, whether following conservative or surgical management, were excluded. Immature skeleton, associated lower limb injuries, previous conditions affecting lower limb function, loss of deep sensation, and systemic illness affecting healing (chronic kidney disease, liver cell failure) or requiring steroid administration were excluded.

Demographic data are presented in Table 1. The level of the rupture was measured intraoperatively with the ankle in the neutral position, median of the distance from the calcaneal insertion at the upper edge of the calcaneal tuberosity was 4.00 cm with IQR (3.38, 5.00).

**Table 1:** Patient’s demographics

Gender	Male:17 (70.8%) Female:7 (29%)
Side (Rt/Lt)	Right:16 (66.6%) Left:8 (33.3%)
Age:	Median: 50 years IQR (40–58)
Mode of injury	6 (25%) during sports 18 (75%) after a fall
Smoking	10 patients (41.6%)
Diabetes (type 2)	6 patients (25%)*
Distance from insertion	Median: 4 cm IQR (3.38-5 cm)
Gap Size	Median: 1.6 Cm IQR (1.2–2.6 cm)
Time to surgery	Median 41 days IQR 36–51
Follow-up period	14.85 months ± 3 months

* all patients had an HbA1c% of 6.5% or lower at the time of surgery

Examination on presentation revealed palpable defect, Thompson and Matles tests were used to assess the Achilles tendon, although less visible than acute cases. We evaluated the patients by radiographs to exclude bony injury, and MRI confirmed the diagnosis. Informed consent was obtained from all cases prior to surgery. This study was conducted per the ethical standards of the Declaration of Helsinki and approved by the ethical committee in our institute. All cases were operated by the same surgeon, using the same technique and rehabilitation protocol. Functional (subjective) outcome of the surgery was assessed using the Achilles tendon Total Rupture Score (ATRS) [15] and the American Orthopedic Foot and Ankle Society (AOFAS) score [16, 17], while the objective assessment was done using the percentage of heel rise comparison to the normal (Heel-rise height index or HRHI) side, and side to side difference of calf circumference.

Data were documented before surgery, six months after surgery and at one year follow-up, documentation was done by an independent orthopedist. Follow-up visits were scheduled as follows: first visit at two weeks for suture removal and splint change from equinus to plantigrade position, second visit was scheduled at four weeks postoperatively for splint removal and initiating range of motion and another visit at eight weeks to initiate weight-bearing. Return to full activity was permitted after 12 weeks. MRI was obtained for the first three cases at one-year postoperative.

**Fig. 1:**
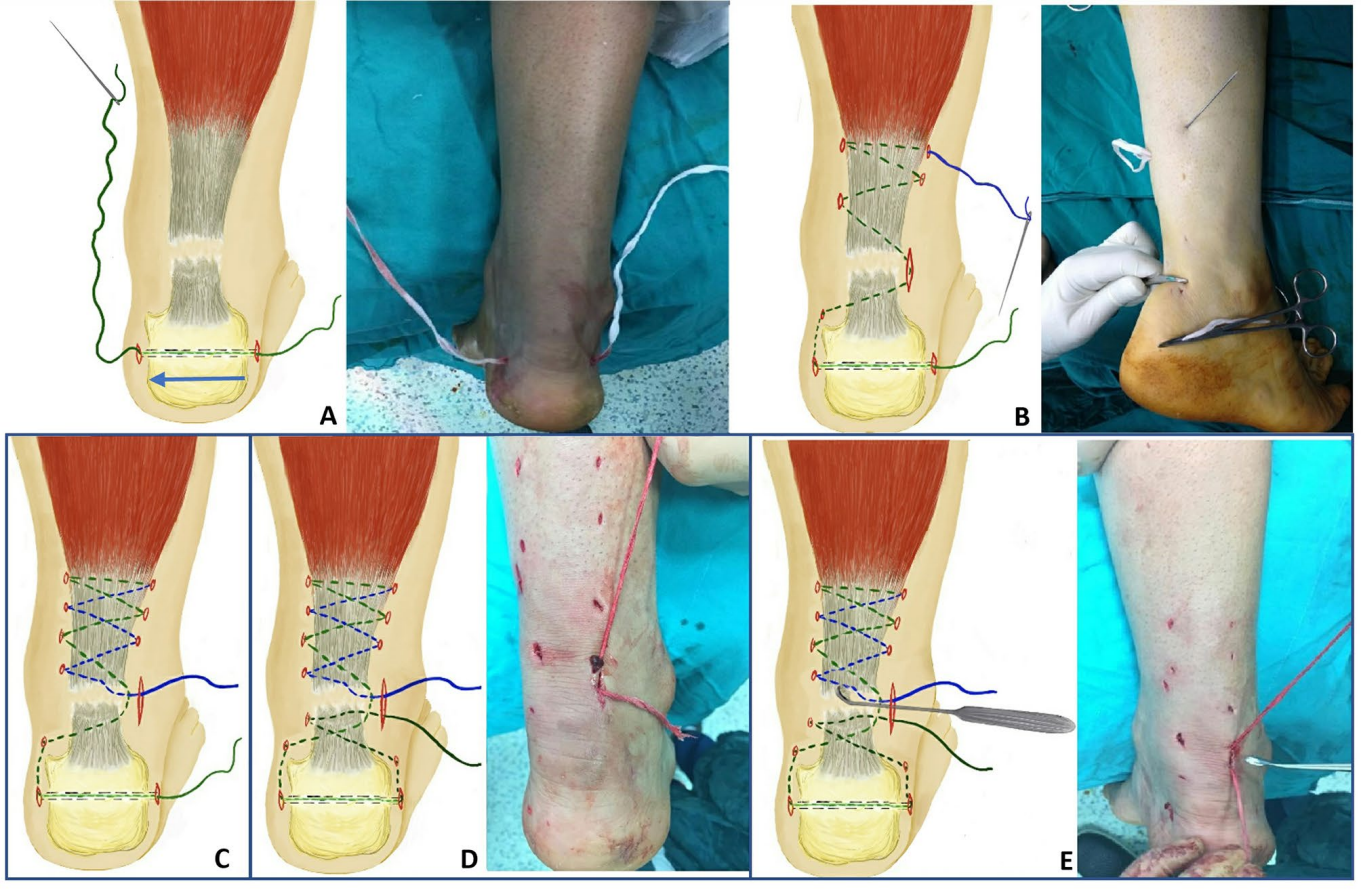
Steps of the surgical Technique **A**: a calcaneal tunnel is made; a non-absorbable suture was passed from lateral to medial. **B**: The suture was passed from medial to lateral, though the substance of the distal stump and exiting at the gap, then directed back medially, and then passed in a zigzag manner from lateral to medial, usually three suture turns. **C**: Then the suture was directed back in a zigzag (blue dotted line) so that the final exit would be through the lateral wound made at the level of the gap. **D**: The distal lateral loose end was then passed under the skin to the level of the Achilles insertion, then passed from lateral to medial through the substance of the distal stump, then back laterally to exit through the lateral wound where the final knot is made. **E**: A sharp curette was then passed through the wound for refreshment of the tendon edges, maximum planter flexion, and the suture is tightened until no gap is palpable

Surgical technique: All cases were operated in a prone position. A bolster was used under the distal part of the leg with the foot hanging off the edge of the table; repair was done using a straight needle loaded with a 5 mm non-absorbable polyester fiber suture (MERSILINE ®, New Brunswick, NJ, USA). The procedure started by making a tunnel in the calcaneus (made to augment the distal hold, and this is done in all cases regardless the size of the distal stump), at the level between distal two thirds and proximal one-third of the calcaneus, then a non-absorbable suture was passed from lateral to medial, advancing to the medial side leaving the free edge on the lateral side (Fig. 1A).

The medial end was then passed subcutaneously to the level of insertion of the Achilles tendon, where a skin snip was made, and threaded needle was retrieved. Through the snip, the suture was then passed from medial to lateral, passing through the substance of the distal stump and exiting at the gap through an incision made over the lateral edge, then directed back medially, catching hold in the proximal fragment and again retrieved through a snip in the skin, and then passed in a zigzag manner from medial to lateral. The soft tissue with each snip was dissected using a mosquito forceps to free the thread from the subcutaneous tissue, and needle is passed directly adjacent to Achilles to avoid sural nerve entrapment, this is continued proximally to a distance allowing three suture turns, usually 10 cm proximal to the gap (Fig. 1B). Then the suture was directed back in a zigzag (blue dotted line in Fig. 1C), directing the needle so that the final exit would be through the wound made laterally at the level of the gap. The loose lateral end was then threaded on a needle and passed subcutaneously to the lateral side of Achilles tendon insertion, then passed from lateral to medial through a snip made proximally at the level of Achilles insertion, passing through the substance of the distal segment of the Achilles tendon from lateral to medial and then passed back laterally to exit through the lateral wound where the final knot is made. (Fig. 1D).

A sharp curette was then passed through the wound for the refreshment of the tendon edges (Fig. 1E), maximum plantar flexion is then made, and the suture was knotted using surgeons knot and tightened until we would no longer be able to palpate a defect by holding the tendon from side to side and direct pressure dorsally, then the knot was held in place using a needle holder and locked with three additional throws. Clinically evident improvement in Thompson and Matles tests was seen post repair. The number of sutures in the distal stump ranged from one to two based on the available distal segment.

A postoperative below-knee splint in resting equinus position was applied for two weeks, then exchanged with another below-knee splint in a plantigrade position for an additional two weeks. A total of one-month splinting is usually needed; oral anticoagulants were given for DVT prophylaxis during this period.

**Fig. 2:**
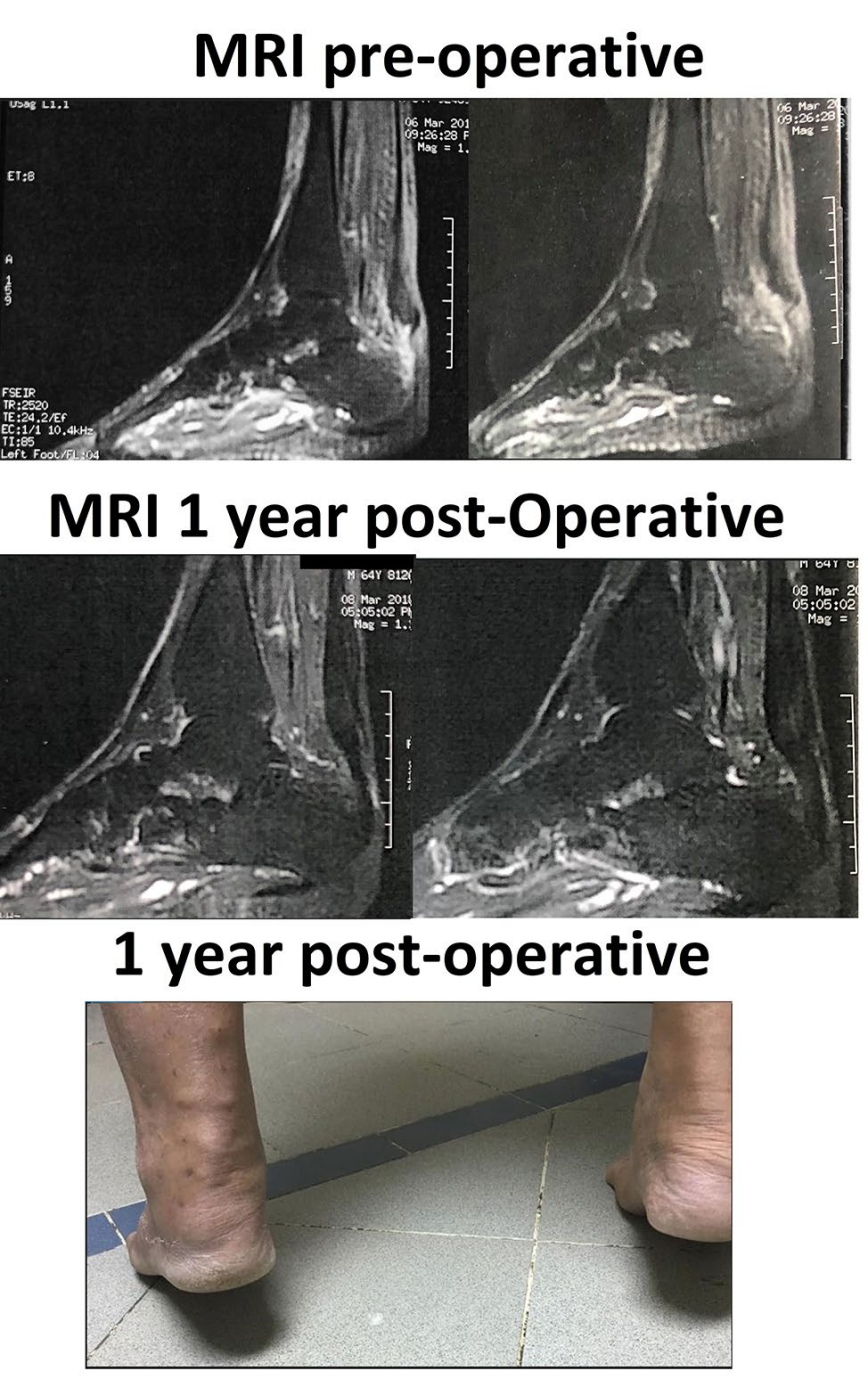
Preoperative and Follow-up MRI at one year - Clinical evaluation at one-year follow-up

Range of motion exercises was initiated afterwards, partial weight-bearing was allowed at eight weeks post-surgery, and progress to full weight-bearing after three months. MRI was performed in 3 patients one year after surgery, showing continuity of the Achilles tendon. (Fig. 2).

We performed the data Analyses using RStudio 1.4.1106 running R 4.0.5. The data distribution was checked using normality tests, plots, and boxplots. We found the data to have non-normal distribution. We presented the continuous data as median (interquartile range -IQR-). The Kruskal-Wallis rank-sum test was used to compare the three time points (preoperative, 6-month, and 12-month follow-up), then we performed post hoc analysis using Dunn’s test as demonstrated in the boxplots.

## Results

**Fig. 3:**
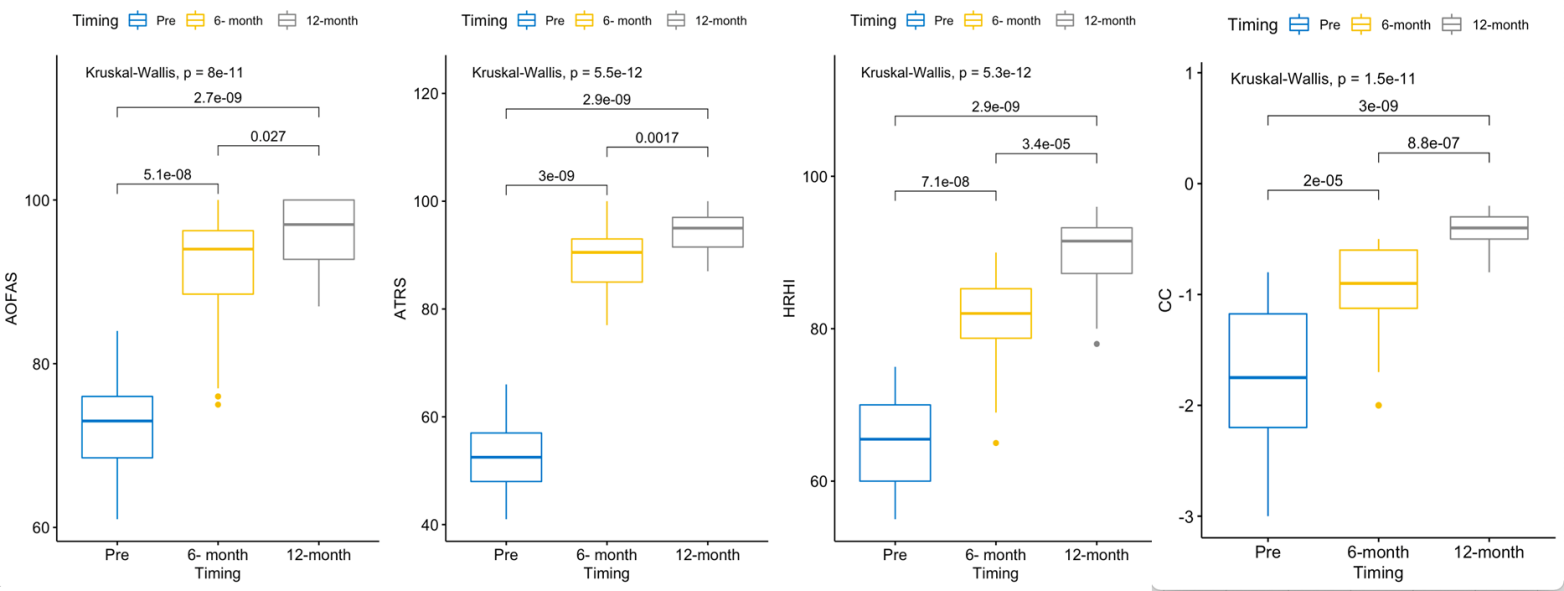
Box plot representing preoperative, 6- and 12-month post-operative for AOFAS, ATRS, heel rise index and calf circumference

Subjective outcome with the median AOFAS at 12 months was 97 (IQR 93, 100), while Median ATRS at 12 months 95 (IQR 92, 97). There was a statistically significant improvement between the preoperative and the post operative AOFAS and ATRS at 6- and 12-months follow-up. Objective outcome with Calf circumference and Heel-rise height index also showed statistically significant difference in preoperative and postoperative scores at 6 and 12 months. (Fig. 3 -Table 2).

Diabetes and smoking had no statistically significant effect on the outcome, probably due to the small sample size. Superficial surgical site infection (SSIs) at one or more of the stab skin incisions was reported in two patients (8.3%), while transient sural nerve neuritis was also reported in two patients (8.3%).

**Table 2:** Results of different subjective and objective parameters

	Timing			p-value^*2*^
	**Pre**, N = 24^*1*^	**6- month**, N = 24^*1*^	**12-month**, N = 24^*1*^	
**AOFAS**	73 (68, 76)	94 (88, 96)	97 (93, 100)	**< 0.001**
**ATRS**	52 (48, 57)	90 (85, 93)	95 (92, 97)	**< 0.001**
**HRHI**	66 (60, 70)	82 (79, 85)	92 (87, 93)	**< 0.001**
**CC**	-1.75 (-2.20, -1.17)	-0.90 (-1.12, -0.60)	-0.40 (-0.50, -0.30)	**< 0.001**

1 Median (IQR)

2 Kruskal-Wallis rank sum test

HRHI: Heel-rise height index

## Discussion

Chronic Achilles ruptures are offered a myriad of reconstruction options, with different types of flaps and grafts which are relatively invasive, with a wide spectrum of complications, not only for Achilles tendon but also for the function of foot and ankle, as well as possible donor site morbidity [18, 19].

Consequently, percutaneous repair of neglected cases is considered an appealing option. It represents a balance between the more invasive reconstructive methods and the simple conservative treatment, offering less operative time, less infection risk, less cost, and more preferred cosmesis [20] Moreover, it does not burn the bridges for other procedures in case of failed repair.

The functional results of the current study were comparable to that of Bertelli et al. [8]. They also used a minimally invasive technique to repair chronic Achilles rupture in twenty patients and achieved satisfactory AOFAS results, with no recorded cases of tendon re-rupture.

Other techniques of managing neglected Achilles tendon rupture have been reported, SAR Ibrahim [21] used a synthetic ligament through open posterolateral approach, AOFAS average 85 ± 6. Ozan et al. [5] reported a more invasive midline posterior incision, with Vulpius lengthening and suturing the tendon ends using modified Kessler. They reported no complications, or infections. However, long midline incision for the Achilles in our experience and literature reports were relatively high. [22].

C. Biz et al. [23] compared two other techniques of percutaneous repair in acute Achilles tendon rupture (within two weeks). Both groups had average final AOFAS of 90 and 90.1, and an ATRS score of 90 and 91, also calf circumference difference was 2.9 cm. Although these results are slightly below ours, this probably because their studied population was recreation athletes specifically. It shows a comparable result for percutaneous repair in both acute (two weeks) and delayed cases.

Different surgical techniques for minimally invasive Achilles tendon repair have been used in literature. C. Biz et al. [23] compared Ma- Griffith with the Tenolig technique in acute cases with results comparable to ours. Carmont et al [24] used 4 modified Bunnel for proximal part, while 4 midified Kessler sutures for the proximal part in acute cases, patients achieved ATRS score of 87 ± 15 and an HRHI 82%±16% at 12 months follow-up.

Maffulli et al. [20] recently reported no difference in the minimally invasive repair of Achilles tendon ruptures on a delayed basis (14–30 days) compared to acute repair at one-year follow-up. Bakowski P. et al. [25] showed promising results in a biomechanical study to analyze the results of minimally invasive repair of Achilles tendon rupture, including one patient with a chronic tear of five years duration.

Pairing these results with the Fact that Healing of Tendoachilles rupture passes through a stepwise procedure to recover (1) inflammatory phase, (2) Fibroblast proliferation and extracellular matrix production and lastly (3) Remodeling phase, with variability its duration, ranging from several weeks up to years, during which, the gap in between the ruptured tendon ends is usually filled with haphazardly organized collagen fibers to bridge the gap between tendon ends [26], It is reasonable to conclude that neglected Achilles ruptures, can seek the secondary intervention at different time points during the remodeling phase.

Although curettage may not remove all the scar tissue with our technique, it was mainly used to debulk the interposed scar tissue. Moreover, it was found that this scar tissue can form tendinous tissue. Thus, incorporating part of the scar tissue in the repair can negate the need for augmented repair and allow a form of delayed primary closure and healing by tertiary intention [27].

Bertelli et al. [8] and Maffuli et al. [20] used absorbable suture (Vicryl). However, as we did our repair in cases longer than four weeks, with a gap distance that averaged 18.6 mm, thus we preferred to use non-absorbable Suture material. We utilized a bone tunnel in the calcaneus regardless of the size of the distal segment, to provide a construct that can withstand the strains applied when pulling the tendon ends together and throughout the tendon healing process.

Sural nerve affection was mitigated by placing the proximal lateral incision just medial the tendon’s lateral border, which helped avoid sural nerve-related complications in 91.6% of the patients. Additionally, all the encountered complications, as sural nerve neuritis and superficial surgical skin infections, were only temporary within the early postoperative period, and completely resolved at the six-month follow-up visit. Which is comparable to the findings by other reports of percutaneous repair [8, 20].

In our cohort, all the patients started full weight-bearing at 3 months which is relatively delayed in comparison to conservative methods [28–30], other minimally invasive methods [8] and open surgical techniques [31]. This cautious approach in rehabilitation has proven beneficial in the long-term results with statically significant steady improvement over time in all the outcome measures used in the current study.

Although there is mounting evidence of comparable results of conservative treatment, it’s beyond the scope of this study, since most conservative treatments are employed in closed acute ruptures, contrary to our study which addresses patients with neglected ruptures [32]. This study provides a reasonable solution to patients who present late, misdiagnosed, or failed conservative treatment. Even though we don’t have the complete data for a long follow-up, we didn’t encounter any cases of re-rupture during the first year.

Reito et al. [30] reported a 7.1% re-rupture rate during the first year in a group of patients treated by functional rehabilitation. In a metanalysis of randomized controlled studies, it was found that conservative treatment of acute rupture had a higher number of re-rupture (9.8%) in comparison to surgical repair(3.7%) [33]. Soroceanu et al. [32] concluded that surgical treatment could be better than conservative treatment if the early range of motion protocols would not be applied.

Yang et al. [12], showed a higher infection rate in the open repair group (3.6%) versus only 0.6% in the percutaneous group, which similar to that reported by F. Olivia et al. [34] in patients with dysmetabolic diseases. Indicating that Percutaneous repair is an appealing option whenever possible. Our reported incidence of infection (8.3%) is relatively higher than the reported literature; however, it could be due to the slightly older age of our cohort (median 50 years – IQR 40–58) as well as high percentage of smoking (41.66%) and diabetes (25%) [35].

The current study is limited by the small number of cases, relatively short-term follow-up, no control with a group of open surgical repair. Also, this study is a retrospective one with inherent bias related to the nature of the study. Secondly, ultrasound was not used during patient management, which would have allowed a more detailed objective assessment of tendon condition preoperatively and during the follow-up period. However, the routine MRI mitigated this for all the included patients before surgery and a few patients postoperatively. On the other hand, the present study has some strength regarding reporting subjective and objective outcomes.

In conclusion, percutaneous repair with a minimally invasive debulking of the interposed scar tissue, paired with a trans-osseous calcaneal tunnel, provides a satisfactory subjective and objective outcome in the management of neglected Achilles tendon rupture, with an accepted degree of complications, and it does not burn the bridges for other potential surgical procedures in the future. We recommend More research regarding the percutaneous management in neglected Achilles tendon rupture with longer follow-up periods.

## Data Availability

The datasets used and/or analysed during the current study are available from the corresponding author on reasonable request.
